# Relationship Between Food Healthiness, Price Fairness, and Loyalty with Moderating Roles of Temperature, Personalization, and Eco-Friendly Packaging at Subway

**DOI:** 10.3390/foods15050841

**Published:** 2026-03-03

**Authors:** Kyung-A Sun, Joonho Moon

**Affiliations:** 1 Department of Tourism Management, Gachon University, Sungnam-si 13120, Republic of Korea; kasun@gachon.ac.kr; 2 Department of Tourism Administration, Kangwon National University, Chuncheon 24341, Republic of Korea

**Keywords:** food marketing, food healthiness, price fairness, loyalty, temperature, personalization, eco-friendly packaging

## Abstract

The purpose of this research is to explore the relationships among food healthiness, price fairness, and loyalty in the context of Subway sandwich restaurants. Another objective of this study is to examine the moderating effects of temperature, personalization, and eco-friendly packaging on the relationship between food healthiness and loyalty. To achieve these objectives, an online survey was conducted. Data were analyzed based on 283 valid responses collected from consumers in the U.S. market. The findings indicate that food healthiness positively influences both price fairness and loyalty. In addition, price fairness exerts a positive effect on loyalty. Furthermore, the results empirically confirmed the significant moderating roles of temperature, personalization, and eco-friendly packaging. This research holds significance in that it empirically clarifies the relationships among the six variables through the case of Subway, providing meaningful marketing insights into consumer perceptions and loyalty in the food service industry.

## 1. Introduction

According to *QSR Magazine* [1], the number of Subway sandwich stores in the U.S. market decreased from approximately 27,000 locations in 2015 to around 17,000 stores in 2024. This decline indicates that the Subway brand is currently facing a challenging situation in the U.S. market. Subway [2] documented that the strength of its menu lies in its low-calorie offerings and its potential to promote health. This health-oriented positioning is likely to provide a comparative advantage over competitors such as McDonald’s in the franchising-based food service sector, particularly in terms of enhancing consumers’ perceptions of health benefits. Consistent with this view, prior research has emphasized that perceived food healthiness plays a critical role in shaping consumers’ decision-making processes and fostering positive product evaluations [3,4,5]. Prior research has claimed that it is inherently constrained by its adverse health implications, while fast food offers advantages primarily in terms of affordability [6,7]. Therefore, the characteristic of Subway sandwiches as a form of fast food that promotes health can be regarded as a unique positioning in the market. In this context, it is both timely and relevant to investigate whether Subway’s marketing strategy—positioning itself as a fast-food brand that emphasizes health—effectively resonates with consumers and addresses prevailing market demands. Such an analysis can be considered worthwhile, as it may offer potential solutions to address the challenges facing Subway’s increasingly weakened market position. From this perspective, the present research explores the health aspects of Subway sandwiches.

This study employed loyalty as the dependent variable. Loyalty has been extensively examined in prior research because it is directly linked to firms’ revenue growth [8,9,10], underscoring its significance for consumer behavior. Mulyawan et al. [11] and Singh et al. [6] alleged that, in franchising-based, high-volume restaurants such as fast-food establishments, rigorous management of customer loyalty is essential in enhancing firm performance. Furthermore, the current study considered price fairness as a mediating variable. Researchers alleged that perceptions of fair pricing arise when products satisfy consumer needs [6,12,13], and competitively priced products encourage consumers’ repeated purchases [11,14].

Next, this research employed the value dilution effect as a central theoretical lens to examine the proposed moderating effects. The value dilution effect documented that when multiple benefits or attributes are simultaneously highlighted, consumers’ cognitive resources are distributed across these cues, leading to a dilution of attention toward the product’s primary value [15,16]. As a result, the focus of the core attribute is likely to be weakened, particularly in consumption contexts where a dominant value proposition exists. In the context of Subway sandwiches, marketing communications frequently emphasize a wide range of food-related attributes, as researchers have addressed, including temperature control [17,18], menu personalization [19,20], and eco-friendly packaging [21,22]. While each of these attributes is likely to independently generate positive evaluations, an excessive or simultaneous emphasis on such peripheral benefits may inadvertently obscure consumers’ perceptions of Subway’s core value proposition—namely, health promotion. Thus, consumers are likely to allocate greater evaluative attention to experiential, functional, or ethical cues rather than to the health-related benefits that fundamentally differentiate the brand. Drawing on this theoretical underpinning, this work posits that temperature, personalization, and eco-friendly packaging function as boundary conditions in the relationship between perceived food healthiness and customer loyalty. Specifically, these attributes are conceptualized as moderating variables that are likely to dilute the effect of food healthiness on loyalty by diluting consumers’ focus on health as the dominant evaluative criterion.

In summary, the purpose of this study was to examine the relationships among food health, price fairness, and loyalty among Subway sandwich consumers. A further objective is to empirically test the moderating roles of temperature, personalization, and eco-friendly packaging. The study contributes academically by extending the understanding of the relationships among food health, price fairness, and loyalty using the Subway case and by clarifying the functions of the moderating variables. In particular, this study provides empirical evidence regarding the explanatory power of the value dilution effect among Subway sandwich consumers. From a practical perspective, the findings offer meaningful insights for sandwich store managers by identifying how marketing resources can be more strategically allocated to enhance brand value and support sustainable brand management.

## 2. Review of the Literature and Proposal of the Hypotheses

### 2.1. Loyalty

Loyalty refers to maintaining a relationship between consumers and sellers as an emotional bond, which is related to the sales growth and higher market share as a sort of performance indicator in the marketing literature [9,23]. Therefore, numerous scholars explored loyalty as a dependent variable because it is associated with the profits of the business [9,24,25]. For instance, Elansari et al. [26] and Kim and Yang [27] employed loyalty to explore the banking service users. Yum and Kim [8] unveiled the determinants of loyalty in the domain of the entertainment platform. Hien and Kim [25] adopted loyalty as a dependent variable to inspect the consumer behavior of agricultural products. García-Salirrosas et al. [10] investigated the antecedents of the loyalty of healthy food products. Also, Arli et al. [24] identified the determinants of loyalty in the area of food delivery application system users. Thus, it can be inferred that multiple studies researched loyalty using a dependent variable in both service and food consumption cases.

### 2.2. Price Fairness

Price fairness is defined as how consumers rationally assess the offered price [6,28]. Adequate price leads consumers to make a decision more easily because of the lower burden, implying that a fair price is essential from the consumers’ point of view. Indeed, many works chose price fairness as a focal attribute. Previous research demonstrated that price fairness was influenced by various attributes and exerted a significant impact on consumer decision-making [12,13]. Singh et al. [6] stated that price fairness was shaped by multiple determinants and functioned as a key element in consumers’ decision-making within the fast-food restaurant context. Heidary and Pluut [29] contended that consumers assessed price fairness based on their perceived utility of products, and such perceptions substantially influenced their purchase decisions. Riquelme and Román [30] found that the presentation of inappropriate prices adversely affected the decision-making processes of online consumers. These findings suggested that price fairness served as both an antecedent and a consequential factor in consumer behavior.

### 2.3. Food Healthiness

In the context of consumer behavior, food healthiness refers to the extent to which consumers perceive food as beneficial to their personal health, taking into account factors such as nutritional value, calories, fat and sugar content, and freshness [3,5]. Chan and Zhang [31] defined food healthiness as consumers’ evaluation based on multiple considerations, noting that such judgments tend to rely on the visible appearance of food and the information provided for consumption. Huang and Lu [32] pointed out that food healthiness plays an important role in enhancing consumers’ purchase intentions. Samoggia et al. [14] found that, among tomato consumers, the perceived health-promoting characteristics of food positively influence perceptions of price fairness. Konuk [33] claimed that growing attention to health promotion in the market has led consumers to place greater value on and spend more for health-beneficial foods.

### 2.4. Hypothesis Development

Jiao et al. [7] alluded that consumers tend to hold skeptical evaluations of fast food from a health perspective, as fast food is characterized by the provision of foods that are high in calories and contain ingredients that elevate cholesterol levels. Mukaromah [34] stated that aspects related to food healthiness make consumers perceive food prices as more reasonable. Konuk [35] demonstrated that, among customers of organic food restaurants, food quality with the potential to influence health positively built consumers’ perceptions of price fairness. Sun and Moon [4] confirmed that the perception of healthy food played a significant role in increasing consumer loyalty. Anesbury et al. [36] contended that the sugar content of food, which is related to the negative effect on health condition plays a key role in shaping consumer loyalty, implying that food healthiness is an important factor in the formation of consumer loyalty. Yoo et al. [37] revealed through consumer exploration that food healthiness is crucial in motivating consumers to repeatedly visit and purchase from specific grocery stores. Based on the theoretical review, it can be inferred that perceptions of food healthiness are likely to influence consumers’ price perceptions and the formation of loyalty. Subway [2] documented that the brand’s market strength is derived from offering consumers healthy menu options. However, research investigating the consumer characteristics associated with this perception remains limited, focusing on the case of the Subway sandwich. Based on these previous studies, the following hypotheses are proposed.

**Hypothesis 1** **(H1).** *Food healthiness positively affects price fairness.*

**Hypothesis 2** **(H2).** *Food healthiness positively affects loyalty.*

Hride et al. [38] reported that price fairness exerted a positive influence on consumer loyalty among online shoppers. Kim and Moon [28] demonstrated that price fairness had a significant positive effect on loyalty among egg consumers. Samoggia et al. [14] confirmed the favorable influence of price fairness on loyalty among tomato consumers, and Mulyawan et al. [11] provided empirical evidence that price fairness significantly contributed to the formation of loyalty among local fast-food restaurant customers. QSR [1] reported that Subway has more locations in the U.S. than McDonald’s, suggesting that the reduction in store numbers was intended to improve the management of its extensive network. This raises the possibility that, due to challenges in maintaining consistent quality across many outlets, Subway may be providing products that fall short of consumer expectations, which could consequently lead consumers to question the value and pricing of its offerings. Based on these findings, the following research hypothesis was proposed:

**Hypothesis 3** **(H3).** *Price fairness positively affects loyalty.*

### 2.5. Value Dilution Effect and Moderating Roles of Temperature, Personalization, and Eco-Friendly Packaging

The value dilution effect refers to a phenomenon in which the impact of existing benefits becomes weakened when consumers are exposed to multiple marketing messages simultaneously [39,40]. Scholars claimed that because the value dilution effect reduces marketing efficiency, providing consumers with multiple benefits at the same time leads to inefficient use of marketing resources [15,16]. Bertin et al. [41] also addressed that, in the context of tomato consumption, presenting consumers with various pieces of information about the advantages of tomatoes simultaneously is likely to diminish the effectiveness of previously perceived benefits.

The temperature of food is critical in the consumption experience, as inappropriate temperatures diminish the perceived flavor and texture of the food [42,43]. Prior works noted that food temperature is an essential cue to appraise food quality [17,18]. Accordingly, scholars contended that food temperature constituted a critical factor that ought to have been considered from the consumer’s perspective [44,45]. Previous research further showed that inadequate temperature conditions served as cues prompting consumers to infer that food had been prepared a considerable time earlier, thereby functioning as a signal that compromised perceptions of food safety [45,46]. These findings collectively indicated that food temperature influenced multiple dimensions of consumer evaluation, including perceived taste and safety. Hence, consumers’ heightened awareness of temperature in food consumption is likely to function to diffuse their value focus on food healthiness, thereby weakening the salience of health-related considerations. Furthermore, personalization denoted the consumer’s capacity to modify the composition of food in accordance with individual preferences [19,20,47]. For instance, Subway sandwiches offered consumers a variety of choices—such as meats, vegetables, bread, and dressings—thereby facilitating customized consumption experiences [21,22]. Scholars stated that individuals were required to engage in a series of decision-making processes to obtain food consistent with their personal preferences, which could deplete their cognitive resources [48,49,50]. Based on this reasoning, personalization might attenuate consumers’ attentional focus on the health-related attributes of food consumption. Lastly, eco-friendly packaging was acknowledged as an instrument that contributed to reducing environmental pollution while simultaneously ensuring the preservation and safety of food by mitigating exposure to harmful substances [51,52]. Extant literature additionally documented that eco-friendly packaging was closely associated with consumers’ perceptions of food safety [53,54]. This implied that the adoption of eco-friendly packaging might redirect consumer attention from concerns about food health toward considerations of food safety. In light of these characteristics from the consumer perspective based on the value dilution effect, the present research formulated the following hypotheses:

**Hypothesis 4** **(H4).** *Temperature significantly moderates the relationship between food healthiness and loyalty.*

**Hypothesis 5** **(H5).** *Personalization significantly moderates the relationship between food healthiness and loyalty.*

**Hypothesis 6** **(H6).** *Eco-friendly packaging significantly moderates the relationship between food healthiness and loyalty.*

## 3. Method

### 3.1. Research Model and Measurement Items

**Figure 1:**
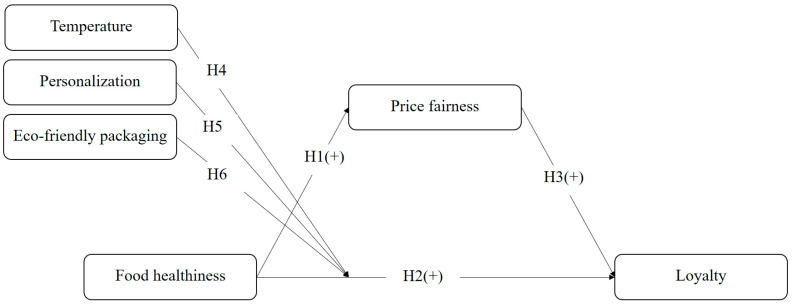
Research model.

Figure 1 displays the research model. Food healthiness exerts positive effects on both price fairness and loyalty. Price fairness positively affects loyalty. Temperature, personalization, and eco-friendly packaging moderate the relationship between food healthiness and loyalty.

Table 1 exhibits the measurement items. This research used Likert five-point scales (1 = strongly disagree, 5 = strongly agree). All the constructs consist of four items. This work referenced the prior works to derive the measurement items, and then the measurement items were adjusted to be more suitable for the objective of this work. The following is the operational definitions of the variables:

Food healthiness: The extent to which consumers perceive Subway sandwiches as beneficial to their health.

Price Fairness: Perceptions of the fairness of Subway’s product prices.

Loyalty: The intention to continue using Subway’s products.

Temperature: Perceptions of the appropriateness of the temperature for consuming Subway’s products.

Personalization: Perceptions of consuming Subway’s products in a way that suits individual preferences.

Eco-friendly Packaging: Perceptions of the environmental friendliness of Subway’s packaging materials.

### 3.2. Recruitment of Survey Participants and Data Analysis Instruments

This study conducted an online survey administered through the Google Survey platform. Participants were recruited using the Clickworker platform service (http://clickworker.com). The Clickworker platform has been widely utilized in academic research for statistical inference purposes [54,55], and its extensive application in prior studies supports the credibility and reliability of the collected data. Given that Subway brand products are publicly accessible, random online sampling was employed. Data collection was conducted between 12 July and 16 July 2025, yielding 283 valid responses, which is considered sufficient to support reliable statistical inference. This work focused on consumers in the US market because Subway is an American-based brand. It enabled the participants to respond to the questions based on their vivid experience because they are more likely to be familiar with the Subway brand. Also, QSR [1] reported that Subway is experiencing challenges in the U.S. market. To explore the underlying reasons for this situation, it was necessary to examine U.S. consumers, and therefore, this research focused specifically on the American consumer market. As Subway is a foodservice brand accessible to a broad consumer base, this study did not impose stringent eligibility restrictions on survey participation to more accurately capture market responses. In addition, by focusing on a specific brand—Subway—this study enabled respondents to more accurately reflect their own consumption experiences when answering the survey questions. Nevertheless, to address ethical considerations, minors were excluded from the survey, and the study was conducted exclusively with respondents aged 20 years and older. Hair et al. [56] contended that a minimum of ten participants per measurement item is required to ensure reliable statistical inference. Furthermore, for structural equation modeling, a sample size exceeding 200 is generally considered adequate to produce stable and robust parameter estimates [57]. With a total of 283 participants, the present study conforms to both of these methodological standards.

A frequency analysis was conducted to obtain demographic information from the survey participants. This work employed exploratory factor analysis with the varimax rotation method to assess the construct validity of the measurement items. The suitability of the data for factor analysis was evaluated using the Kaiser–Meyer–Olkin (KMO) measure of sampling adequacy, with values exceeding 0.70 indicating adequacy, and Bartlett’s test of Sphericity χ^2^, following the guidelines proposed by Hair et al. [56]. Following the recommendations of Hair et al. [56], convergent validity of the measurement items was evaluated using the following criteria: factor loadings above 0.5, Cronbach’s α coefficients greater than 0.7, and eigenvalues exceeding 1. Correlation analysis was then performed to examine the relationships among the key variables—nutritional value, attitude, repurchase intention, and price fairness—and to compute their respective means and standard deviations. Path analysis was carried out using Hayes’ Process Macro model 5, which employs ordinary least squares regression. According to Hayes [58], the Process Macro minimizes sample distortion and produces more robust estimates, as it is less sensitive to violations of normality. Furthermore, it enables simultaneous estimation of complex mediation and moderation models within a single analytical framework. This research additionally implemented the simple slope method to scrutinize the moderating effects graphically.

## 4. Empirical Results

### 4.1. Profile of the Survey Participants

Table 2 presents the demographic characteristics of the respondents. Females comprised 65.4% of the sample, while participants in their 30s and 40s represented approximately 67.5%. Table 2 also reports participants’ monthly household income (<$2500: 82; $2500–4999: 91; $5000–7499: 45; $7500–9999: 26; ≥$10,000: 39) and terminal academic degree (less than college: 120; bachelor’s degree: 103; graduate degree: 60). Regarding usage frequency, 185 respondents (65.4%) reported using Subway less than once per week.

### 4.2. Validity and Reliability of the Measurement Items and Correlation Matrix

Table 3 presents the results of the validity and reliability of the measurement items. All factor loadings are greater than 0.5. All Cronbach’s alphas are greater than 0.7. The goodness-of-fit indices indicate that the results of the factor analysis are statistically acceptable (KMO = 0.915, and Bartlett’s Test of Sphericity χ^2^ = 8527.571 (*p* < 0.01)). Given the results, this work ensured the validity of the six constructs. Table 3 also includes the means and SDs of the variables: food healthiness (mean = 3.89, SD = 1.08), price fairness (mean = 3.41, SD = 1.07), loyalty (mean = 3.84, SD = 1.16), temperature (mean = 4.15, SD = 0.86), personalization (mean = 4.46, SD = 0.73), and eco-friendly packaging (mean = 3.54, SD = 1.00).

Table 4 is the correlation matrix. All variables are positively correlated. Loyalty exhibits the strongest correlation with food healthiness. Price fairness showed the strongest correlation with temperature (r = 0.505). According to Hair et al. [56], when the absolute values of correlation coefficients are below 0.7, concerns regarding multicollinearity are considered minimal. Since none of the correlation coefficients in this study exceed 0.7, all proposed constructs are retained in the analysis.

### 4.3. Testing Results of Hypotheses

Table 5 depicts the results of hypothesis testing. All four models are statistically significant, given the F-values (*p* < 0.05). Food healthiness is positively associated with price fairness (β = 0.44, *p* < 0.05). Also, food healthiness (β = 0.91, *p* < 0.05) and price fairness (β = 0.25, *p* < 0.05) exerted positive influences on loyalty. Hence, H1, H2, and H3 are supported. The moderating effects of temperature (β = −0.10, *p* < 0.05), personalization (β = −0.12, *p* < 0.05), and eco-friendly packaging (β = −0.07, *p* < 0.1) on the impact of food healthiness on loyalty appeared significant. Thus, H4, H5, and H6 are supported.

**Figure 2:**
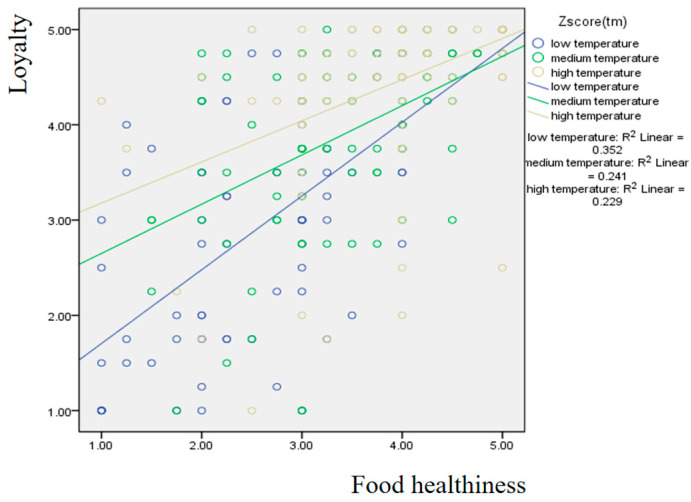
Moderating effect of temperature on the influence of food healthiness on loyalty.

Figure 2 presents the results regarding the moderating effect of temperature. For the group perceiving a lower level of appropriateness in temperature, the slope of the effect of food healthiness on loyalty is the steepest, whereas the group perceiving a higher level shows the smallest slope.

**Figure 3:**
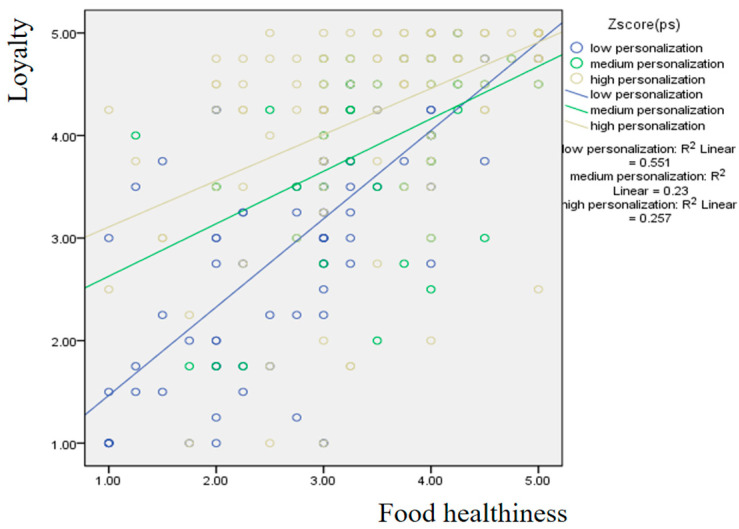
Moderating effect of personalization on the influence of food healthiness on loyalty.

Figure 3 shows the results of the moderating effect of personalization. It can be observed that for the group with a low perception of personalization, the effect of food healthiness on loyalty appears to be the strongest.

**Figure 4:**
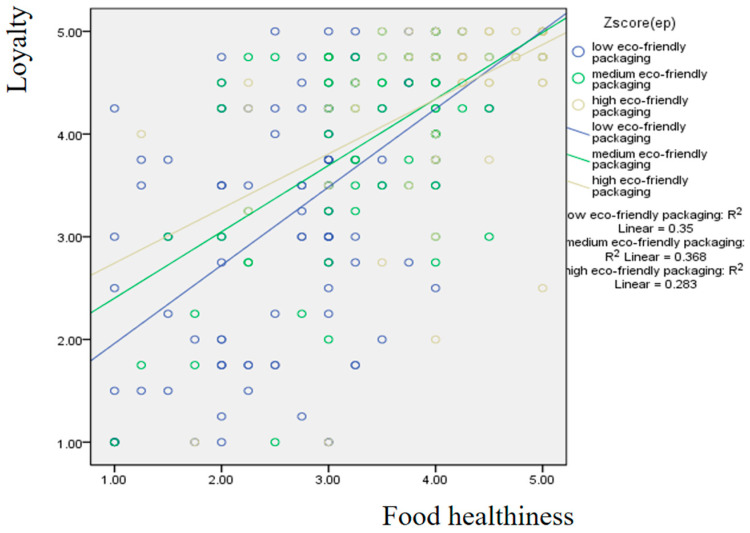
Moderating effect of eco-friendly packaging on the influence of food healthiness on loyalty.

Figure 4 illustrates the results concerning the moderating effect of eco-friendly packaging perception. The group with a lower perception of eco-friendly packaging demonstrates a more moderate effect of food healthiness on loyalty compared to the group with a higher perception.

## 5. Discussion

This work investigated consumers in the U.S. market of Subway sandwich restaurants. The mean values indicated that consumers held the strongest perceptions regarding personalization in their Subway sandwich consumption. The findings likely stemmed from consumers’ familiarity with Subway’s ordering system, which allowed customers to select ingredients such as cheese, meat, vegetables, and bread. Consumers also exhibited relatively high perceptions of temperature, which could be attributed to their recognition of Subway’s differentiated preparation system, in which ingredients intended to be served warm and those meant to remain cold were handled separately. In contrast, consumers demonstrated comparatively skeptical perception toward the healthiness of the food, which might reflect the perception that Subway products continued to be categorized primarily as fast food from the consumers’ perspective.

Hypothesis testing results indicated that food healthiness exerted a significant positive effect on both price fairness and loyalty. Specifically, the provision of food prepared with health-promoting ingredients appeared to reduce consumers’ price resistance and served as an important driver of continued purchasing behavior at Subway. The results of this study corroborated prior research by confirming that health-promoting factors played a significant role in consumers’ food choice decisions [4,37]. Furthermore, the findings confirmed a positive effect of price fairness on loyalty. Given that food was regarded as a necessity, the presentation of a reasonable price level played a critical role in strengthening consumer loyalty, a pattern that was also observed in the Subway context. The findings of this work exhibited a pattern consistent with previous literature, indicating that price fairness played a pivotal role in shaping consumer loyalty within the food consumption context [14,28].

The moderating effect of temperature was found to be significant. The results suggested that when consumers’ loyalty formation related to food healthiness simultaneously involved heightened attention to temperature, the salience of food healthiness was diluted. This outcome could be explained by a dispersion of consumers’ value focus. A similar pattern was observed for personalization, as consumers who customized their sandwiches by adding preferred ingredients might incorporate higher-calorie options, thereby weakening the influence of perceived food healthiness on loyalty. Finally, the study identified evidence of value dispersion associated with eco-friendly packaging. Although eco-friendly packaging was perceived as reducing environmental pollution, it also involved additional considerations related to minimizing harmful substances, such as endocrine disruptors or microplastics, in packaging materials. These multiple value considerations might increase the effect of food healthiness on loyalty. Overall, the findings implied that consumers’ value-related evaluations of temperature, personalization, and eco-friendly packaging dispersed their attention away from food healthiness, thereby diminishing its influence on loyalty. The results of this study corroborated previous literature suggesting that when consumers are presented with multiple pieces of information simultaneously, the perceived benefits of a specific product may be diluted [15,16]. Furthermore, the present study demonstrated that this effect can manifest in relation to factors such as temperature, personalization, and eco-friendly packaging.

This work provides several practical suggestions for managers. First, sandwich shop managers might be able to focus on offering sandwiches that promote health. Highlighting aspects such as calorie content and ingredient quality can help achieve this. Managers also might be able to pay attention to managing suppliers to ensure a stable supply of fresh ingredients, which supports the sustainable operation of the shop. Franchisees might also be able to pay greater attention to inventory management in this regard, as the use of aged ingredients can compromise the freshness of food and potentially have adverse health effects. In terms of online marketing, such as social network marketing, it is necessary to keep in mind that emphasizing marketing strategies that prominently display images emphasizing fresh vegetable ingredients in ways that are highly visible to consumers, and using these strategies to actively communicate with consumers, can also serve as an effective means of enhancing firm performance. Moreover, managers might be able to maintain stable pricing. Sudden or large price changes may reduce consumers’ perception of price fairness and could lead to the loss of loyal customers. Ensuring consistent and fair pricing can help retain customer loyalty. To maintain stable pricing, the franchising headquarters might be able to focus its capabilities on ensuring a consistent supply of ingredients. Finally, managers could be careful about using too many marketing strategies at once. Moreover, when confidence in price competitiveness exists, managers of sandwich franchise chains may achieve a competitive advantage in online marketing by developing advertising materials that emphasize price-related information and delivering them effectively to consumers. Using factors such as temperature, personalization, and eco-friendly packaging together may distract consumers’ attention and make it harder for the intended marketing message to be effectively communicated. In addition, concerning aspects such as food temperature and customization, it could be valuable that employees are well acquainted with operational manuals and that effective communication with customers is ensured. In this respect, for franchised restaurants such as Subway, it can be recommended that firms need to allocate organizational resources to employee training as well as to communication and coordination with franchise owners.

This work has several limitations. First, it focused exclusively on consumers in the U.S. market, which may limit the generalizability of the findings. As Subway is a global food service brand, it is important to incorporate perspectives from more diverse markets to provide broader insights. Future researchers are encouraged to address this limitation by reducing the theoretical gap through cross-cultural or multi-market analyses. Second, the research relied solely on survey-based data, which may restrict the depth of understanding regarding consumer behavior. To overcome this limitation, future work needs to consider employing various methodological approaches—such as experimental designs or qualitative methods—to gain a more comprehensive understanding of consumers’ characteristics and decision-making processes. Although heavy users comprised only 34.7% of the current sample, thereby limiting the statistical power to examine the moderating effects of socio-demographic variables such as income and usage frequency, the present findings nonetheless provide meaningful insights into general consumer perceptions. Future research with a larger or more strategically targeted sample could more rigorously assess these potential moderating effects. Finally, because this study was executed with a specific focus on the Subway brand, there may be limitations in generalizing the findings to other contexts. Future research should address this limitation by examining similar relationships across multiple brands rather than concentrating on a single brand, thereby enhancing the generalizability of the results.

## 6. Conclusions

This work has theoretical implications. First, it provides meaningful insights by clearly identifying the relationships among food healthiness, price fairness, and loyalty through the case of Subway sandwich restaurants. Second, it offers theoretical and practical value by revealing that various marketing elements—such as temperature, personalization, and eco-friendly packaging—may distract consumers’ value focus in the decision-making process. In other words, this work highlights the dispersion effect of value focus, demonstrating how multiple marketing stimuli can weaken consumers’ consistent evaluation of core value perceptions.

## Figures and Tables

**Table 1:** Measurement items description.

Attributes	Codes	Measurement Items	Reference
Food healthiness	FH1	Subway food contributes positively to my health.	Konuk [33] Sun & Moon [4]
	FH2	Subway food is nutritious.	
	FH3	Choosing Subway food can improve my health.	
	FH4	Subway food is beneficial for maintaining good health.	
Price fairness	PF1	The prices at Subway are fair.	Singh et al. [6] Kim & Moon [28]
	PF2	The prices at Subway are reasonable.	
	PF3	The prices at Subway are acceptable.	
	PF4	The prices at Subway are affordable.	
Loyalty	LO1	I am loyal to the Subway brand.	Miguel et al. [23] Pereira et al. [9]
	LO2	I intend to visit a Subway store again.	
	LO3	I plan to purchase Subway products again.	
	LO4	I am willing to make repeat purchases from Subway.	
Temperature	TM1	The temperature of Subway food is appropriate.	Namkung & Jang [17] Kang & Namkung [18]
	TM2	Subway food is served at the right temperature.	
	TM3	The temperature of Subway food meets my expectations.	
	TM4	I am satisfied with the temperature of Subway food.	
Personalization	PS1	Subway food can be customized to my preferences.	Ho [47] Lee et al. [50]
	PS2	I can personalize my food choices at Subway.	
	PS3	Subway offers customized food options.	
	PS4	Subway provides personalized meal options.	
Eco-friendly packaging	EP1	Subway product packaging is eco-friendly.	Sun & Moon [54]
	EP2	Subway product packaging is environmentally sustainable.	
	EP3	Subway product packaging helps minimize waste.	
	EP4	Subway product packaging is environmentally beneficial.	

**Table 2:** Survey participant profile (N = 283).

Characteristics	Frequency	Percentage
Male	98	34.6
Female	185	65.4
20s	46	16.3
30s	98	34.6
40s	93	32.9
50s	39	13.8
Aged > 60 years	7	2.5
Monthly household income		
<$2500	82	29.0
$2500–4999	91	32.2
$5000–7499	45	15.9
$7500–9999	26	9.2
≥$10,000	39	13.8
Terminal academic degree		
Less than college	120	42.4
Bachelor’s degree	103	38.4
Graduate degree	60	21.2
Weekly usage frequency		
Less than 1 time	185	65.4
1–2 times	78	27.6
3–6 times	18	6.4
More than 7 times	2	0.7

**Table 3:** Validity and reliability of the measurement items.

Construct	Code	Loading	Mean (SD)	Cronbach’s α	Eigenvalue	Explained Variance
Food healthiness	FH1	0.787	3.39 (1.08)	0.949	2.676	11.150
	FH2	0.809				
	FH3	0.838				
	FH4	0.828				
Price fairness	PF1	0.889	3.41 (1.07)	0.971	12.021	50.086
	PF2	0.897				
	PF3	0.881				
	PF4	0.903				
Loyalty	LO1	0.554	3.84 (1.16)	0.939	1.008	4.201
	LO2	0.854				
	LO3	0.821				
	LO4	0.823				
Temperature	TM1	0.797	4.15 (0.86)	0.958	1.363	5.679
	TM2	0.821				
	TM3	0.804				
	TM4	0.811				
Personalization	PS1	0.829	4.46 (0.73)	0.929	1.829	7.622
	PS2	0.874				
	PS3	0.890				
	PS4	0.787				
Eco-friendly packaging	EP1	0.885	3.54 (1.00)	0.951	2.209	9.202
	EP2	0.874				
	EP3	0.867				
	EP4	0.880				

Note: SD stands for standard deviation, the unit of explained variance is percent, total variance explained: 87.940%, Kaiser-Meyer-Olkin Measure (KMO) of Sampling Adequacy: 0.915, Bartlett’s Test of Sphericity χ^2^: 8527.571 (*p* < 0.01).

**Table 4:** Correlation matrix.

	1	2	3	4	5
1. Loyalty	1				
2. Price fairness	0.528 *	1			
3. Food healthiness	0.660 *	0.450 *	1		
4. Temperature	0.588 *	0.505 *	0.590 *	1	
5. Personalization	0.519 *	0.358 *	0.400 *	0.588 *	1
6. Eco-friendly packaging	0.410 *	0.400 *	0.543 *	0.421 *	0.319 *

Note: * *p* < 0.05.

**Table 5:** Results of hypothesis testing using Hayes’ Process Macro model 5.

	Model1	Model2	Model3	Model4
	β-Value (t-Value)	β-Value (t-Value)	β-Value (t-Value)	β-Value (t-Value)
	Price Fairness	Loyalty	Loyalty	Loyalty
Constant	1.89 (10.02) **	−1.04 (−1.66) *	−1.95 (−2.58) **	0.02 (0.05)
Food healthiness	0.44 (8.43) **	0.91 (4.20) **	1.03 (3.92) **	0.80 (−3.52) *
Temperature		0.60 (3.81) **		
Personalization			0.73 (4.27) **	
Eco-friendly packaging				0.25 (1.68) *
Food healthiness × Temperature		−0.10 (−2.15) **		
Food healthiness × Personalization			−0.12 (−2.10) **	
Food healthiness × Eco-friendly packaging				−0.07 (−1.75) *
Price fairness		0.25 (4.76) **	0.25 (4.27) **	0.31 (6.04) **
F-value	71.17 **	81.33 **	88.94 **	71.80 *
R^2^	0.2021	0.5392	0.5614	0.5082
Conditional effect of focal predictor		Temperature	Personalization	Eco-friendly packaging
		3.00:0.59 (7.21) **	4.00:0.54 (9.53) **	2.75:0.61 (9.81) **
		4.00:0.48 (8.29) **	5.00:0.42 (7.36) **	3.50:0.55 (9.81) **
		5.00:0.37 (5.45) **	5.00:0.42 (7.36) **	4.75:0.46 (5.94) **
Interaction effect: F-value		4.65 **	4.44 **	3.09 *

Note: * *p* < 0.1, ** *p* < 0.05.

## Data Availability

The data presented in this study are available upon request from the corresponding author. The data are not publicly available due to privacy concerns.
